# Durable clinical benefit to trastuzumab and chemotherapy in a patient with metastatic colon adenocarcinoma harboring *ERBB2* amplification

**DOI:** 10.18632/oncoscience.175

**Published:** 2015-07-01

**Authors:** Umut Disel, Alexis Germain, Bahar Yilmazel, Huseyin Abali, Filiz Aka Bolat, Roman Yelensky, Julia A. Elvin, Doron Lipson, Juliann Chmielecki, Kai Wang, Philip J. Stephens, Jeffrey S. Ross, Vincent A. Miller, Siraj M. Ali, Thomas J. George

**Affiliations:** ^1^ Adana Numune Education and Research Hospital, Department of Medical Oncology, Adana/Turkey; ^2^ Foundation Medicine, Inc. Cambridge, MA, USA; ^3^ Baskent University School of Medicine Adana, Turkey; ^4^ Albany Medical Center, Albany, NY; ^5^ University of Florida College of Medicine Gainesville, FL, USA

**Keywords:** *ERBB2*, HER2, trastuzumab, oxaliplatin, colorectal adenocarcinoma

## Abstract

Somatic *ERBB2* amplification or activating mutations occur in approximately 2–5% of metastatic colorectal adenocarcinomas and are presumed to be oncogenic drivers, but limited evidence exists to suggest these lesions are sensitive to targeted monotherapy in patients. Here we present the case of a patient with advanced CRC with pulmonary metastases, who had progressed on both standard of care cytotoxic chemotherapy and anti-*EGFR* targeted therapy. Comprehensive genomic profiling (FoundationOne^®^) identified amplification of *ERBB2* and a *TP_53_* mutation in the metastatic lesion. Treatment with trastuzumab with a chemotherapy backbone elicited stable disease/minor response in the patient over a one year course of therapy, reducing tumor burden and significantly improving quality of life. This report demonstrates the application of personalized targeted therapy guided by comprehensive genomic profiling in metastatic colorectal adenocarcinoma.

## CASE PRESENTATION

A 39-year old female was diagnosed with a sporadic rectal adenocarcinoma which was excised via low anterior resection (LAR), and staged through pathologic examination of the tumor as pT3N0 without infiltration of adjacent lymph nodes or distant metastasis [1]. The patient received adjuvant chemoradiotherapy followed by 6 months of infusional 5-fluorouracil (5-FU) and leucovorin chemotherapy. Approximately one year after initial diagnosis, two lesions were discovered in the lungs consistent with metastatic disease. Pulmonary metastasectomy was completed followed by a 6 month course of FOLFIRI (5-FU, leucovorin and irinotecan) and bevacizumab. One year later, identification of additional lesions in the lungs prompted a second metastasectomy followed by 6 cycles of capecitabine. Her disease continued to progress with multiple metastases to the lungs, abdomen, and pelvis. To obviate symptoms arising from a large pelvic tumor, abdomino-perineal resection was performed and subsequent molecular testing confirmed the tumor as *KRAS* wild-type. Anti-*EGFR* combination treatment with FOLFIRI and cetuximab was initiated and continued for 9 months until termination due to disease progression. Patient then received XELOX (capecitabine and oxaliplatin) chemotherapy for 7 months again until disease progression occurred. Her ECOG performance status declined to 3 due largely to cancer-related symptoms including limited mobility, dyspnea with cough, and abdominal pain. Comprehensive genomic profiling was obtained to identify rational approaches to targeted therapy in a young patient who had fully exploited guideline driven therapies.

## RESULTS

**Figure 1:**
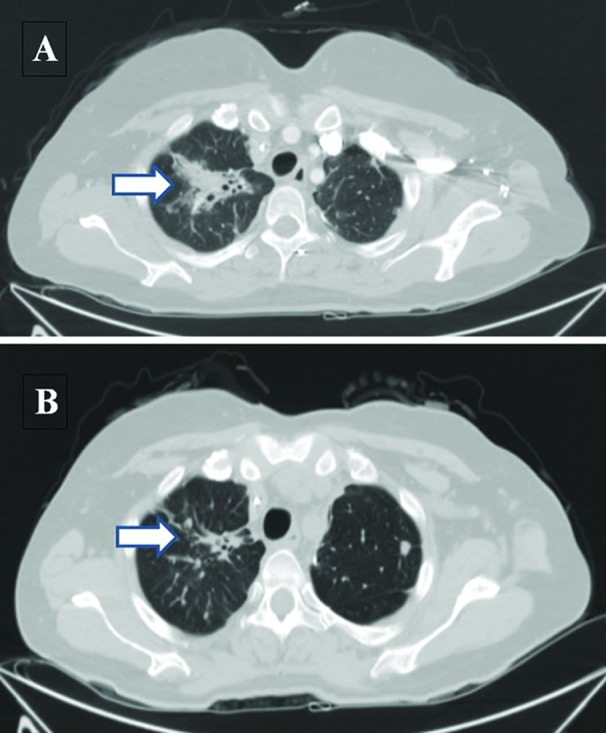
Representative CT scan images of upper lung metastasis at baseline (A) and after 3 months of trastuzumab and chemotherapy (B) Arrow indicates significantly regressed tumor burden. (Note: respiratory variability accounts for slight anatomic differences in pre/post imaging).

Comprehensive genomic profiling identified amplification of *ERBB2* (quantitatively estimated as 21 copies) in the metastatic tumor, as well as a loss of function point mutation within *TP53*. Combination therapy of trastuzumab with a backbone of capecitabine and oxaliplatin was started. The patient's performance status began to improve within two months of initiating treatment, with an ECOG score of 2, improving over the course of the next five months to zero, reflecting increased mobility and near complete resolution of symptoms. Of note, oxaliplatin was eliminated after 6 months of treatment. Imaging with PET/CT scans at 3 and 5 months post anti-*ERBB2* treatment showed reduction of tumor burden with SUV in the lesion at the apex of the right lung falling from 5.1 to 4.0 and reduction of tumor volume by close to 20% (Figure 1). For the residual pelvic disease, SUV reduced from 12.2 to 9.1 with an estimated 33% reduction in metastatic disease in that area. The tumor markers CEA and CA 19-9 both showed marked declines over the first 7 months of treatment, each reduced to a third of their initial values. Treatment with trastuzumab continued for 12 months in total, after which time the patient's symptoms returned with biomarkers and radiology confirming progressive disease.

## METHODS

Metastatic tumor samples from a previous pulmonary metastasectomy were submitted for histopathology, as well as comprehensive genomic profiling using the FoundationOne^®^ assay (Foundation Medicine Inc., Cambridge, MA). Hybridization capture of 3,734 exons from 236 cancer-related genes and 47 introns of 19 genes commonly rearranged in cancer was applied to ≥ 50 ng of DNA extracted from formalin fixed paraffin embedded tumor and sequenced to a median coverage depth of > 500x [2].

## DISCUSSION

Colorectal cancer affects a substantial patient population, with an estimated 132,700 incident cases in the US in 2015. CRC causes 49,700 deaths annually, making this disease the second most common cause of cancer death in men and women combined [3]. Prognosis is tightly linked to the stage at diagnosis, with patients presenting with metastatic disease surviving a median of 2 years with palliative systemic therapy [4]. Recent advances in surgical resection and combination therapy have significantly improved the 5 year survival for a subset of patients with oligometastatic disease [5]. However, for most patients with metastatic disease, medical management is oriented towards minimizing disease and treatment-associated complications in the context of disease palliation [6].

*ERBB2* encodes the receptor tyrosine kinase HER2, a member of the epidermal growth factor receptor family that has been linked to normal cell proliferation and tissue growth. *ERBB2* amplification (2.5–3%) or mutation (~2%) is present in a small subset of CRC patients [7,8]. In CRC, overexpression of membranous HER2 corresponds to the observed frequency of associated gene alterations [9]. Little is known about the prognostic value of HER2 overexpression in CRC, although a recent meta-analysis found no significant effect on survival [10]. *ERBB2* amplification and HER2 overexpression have best been characterized in carcinomas of the breast where it classifies 15–20% of cases as a distinct subset with poor prognosis and increased probability of recurrence [11]. Trastuzumab, an anti-HER2 monoclonal antibody that targets the extra-cellular domain, is FDA-approved for use in gastric and breast cancers overexpressing HER2 and has shown efficacy in combination with cytotoxic chemotherapy [12,13]. Studies of *ERBB2* amplification in gastric and breast cancer patients have shown a correlation between copy number alteration and sensitivity to treatment with trastuzumab, with higher copy number alteration leading to more complete responses and improved overall survival [14,15].

The efficacy of trastuzumab in breast and gastric cancers suggests by analogy that patients with *ERBB2* amplified CRC may also benefit from targeted anti-HER2 therapies such as trastuzumab, as in the case reported here. At least two case studies have demonstrated consistent responses of *ERBB2* amplified left sided colorectal carcinoma and rectal carcinoma to trastuzumab monotherapy [16,17]. The relatively high copy number observed in this case of rectal carcinoma may predict response to anti-HER2 targeted therapy, akin to the same phenomenon in *ERBB2* amplified breast cancer, but quantitative estimates of copy number were not available for the previous cases in the literature.

In a recent report by the HERACLES study investigators, 913 patients with metastatic CRC were screened to identify 44 with HER2 overexpression [18]. Although preliminary, treatment with trastuzumab and lapatinib in this sub group of *ERBB2* amplified cancer as identified by FISH or IHC was without significant toxicity and demonstrated moderate activity. Other clinical trials targeting this population with anti-HER2 targeted therapy and a chemotherapy backbone such as NCT00006015 and NCT00003995 were initiated over a decade ago and had negative results, but enrolled either an unstratified population of advanced CRC patients, or relied on solely IHC to screen in patients [19]. The early termination of these trials due to lack of sufficient accrual has led some to propose limited applicability of anti-HER2 therapy in CRC patients due to the low frequency of HER2 overexpression [20]. However, due to the high incidence of CRC, anti-HER2 targeted therapy has the potential for use in over 3,000 patients a year in the US alone. Comprehensive genomic profiling offers both quantitative estimation of *ERBB2* amplification as well as assessment of the remainder of the genomic profile, and when applied in the course of clinical care could provide greater insight into the efficacy of anti-HER2 targeted therapy in *ERBB2* amplified CRC patients.

The prognosis is unfavorable for patients with metastatic CRC refractory to standard treatments with median survival of approximately 6-9 months [21,22]. In this patient, the additional activating alteration of *TP53* also identified through comprehensive genomic profiling has been associated with poor survival [23]. This patient with an *ERBB2* amplified rectal carcinoma, after exhausting prior guideline driven therapies, responded for 12 months to trastuzumab with a XELOX backbone. Thus, this case illustrates a much better than expected outcome given failure of standard treatments, and in comparison to benefit from prior lines of therapy in the same patient. In contrast to the failure of previous treatment with XELOX alone, this patient's subsequent response suggests that the efficacy was driven by the addition of trastuzumab, likely linked to the relatively high *ERBB2* amplification in this case quantitatively estimated to be 21 copies.

Some investigators have suggested the ratio of progression free survival on a given regimen exceeding that of a precedent one as another surrogate for biologic effect in advanced cancer [24]. The current case of a patient treated with trastuzumab and chemotherapy demonstrates a clear, durable, symptomatic, and radiographic benefit and without significant toxicity after exhaustion of standard of care therapy. Although progression ultimately occurred, the patient experienced substantial improvement in cancer-associated symptoms. This approach of comprehensive genomic profiling allows identification of clinically relevant but infrequent genomic alterations that would otherwise remain unrecognized in the context of traditional molecular testing for colorectal carcinoma. Further investigation both in the setting of clinical trials and routine clinical practice is required to elaborate on these findings.
